# Prevalence and determinants of nasal carriage of penicillin non-susceptible *Streptococcus pneumoniae*: a cross-sectional household survey in northern Vietnam

**DOI:** 10.1016/j.lanwpc.2024.101282

**Published:** 2025-01-08

**Authors:** Costanza Tacoli, Hien Anh Thi Nguyen, Tu Cam Thi Nguyen, Bich Ngoc Thi Vu, Max van Wijk, Quynh Dieu Pham, Huong Kieu Thi Tran, Thuong Hong Thi Nguyen, Trang Thu Nguyen, Tung Son Trinh, Dung Tien Viet Vu, Hoang Huy Tran, Thai Duy Pham, Duc Anh Dang, Tien Dac Tran, Duong Thanh Nguyen, H. Rogier van Doorn, Thomas Kesteman, Sonia Lewycka

**Affiliations:** a Oxford University Clinical Research Unit (OUCRU), National Hospital for Tropical Diseases, 78 Giai Phong, Dong Da District, Hanoi, Viet Nam; b National Institute of Hygiene and Epidemiology (NIHE), 1 Yec Xanh, Hanoi, Pham Dinh Ho, Hai Ba Trung, Viet Nam; c Faculty de Pharmacy – University of Tours, Tours, France; d Centre for Disease Control, Ha Nam Province, Viet Nam; e Department of Health, Ha Nam Province, Viet Nam; f Centre of Tropical Medicine and Global Health, Nuffield Department of Medicine, University of Oxford, New Richards Building, Roosevelt Dr, Headington, Oxford OX3 7LG, United Kingdom

**Keywords:** Antibiotic, Resistance, Penicillin, *Streptococcus pneumoniae*, Commensal, Carriage, Community, Meat, Vietnam

## Abstract

**Background:**

Beta-lactams remain the first-line treatment of *Streptococcus pneumoniae* infections despite the increasing global prevalence of penicillin-resistant/non-susceptible strains. We conducted a cross-sectional household survey in a rural community in northern Vietnam in 2018–2019 to provide prevalence estimates of penicillin non-susceptible *S. pneumoniae* (PNSP) carriage and to investigate behavioural and environmental factors associated with PNSP colonization. The data presented will inform the design of a large trial of population-based interventions targeting inappropriate antibiotic use.

**Methods:**

A survey was conducted between July 2018 and April 2019, encompassing 1502 individuals from 324 households. A total of 1, 498 samples from nasal and nasopharyngeal swabs were cultured on blood agar plates supplemented with 5 μg of gentamicin. *S*. *pneumoniae* colonies were confirmed by MALDI-TOF. Penicillin susceptibility was tested by E-test. Logistic regression models were used to explore risk factors for PNSP carriage compared to susceptible strains.

**Findings:**

We recovered 132 *S. pneumoniae* isolates out of 1148 swabs. Antibiotic susceptibility results were obtained for 97% (128/132). Of these, 76% (97/128) were PNSP (MIC ≥ 0.12 μg/ml) and 77% (99/128) were non-susceptible to three or more antibiotics. After adjusting for age and wealth, antibiotic use was not associated with PNSP carriage. Participants more likely to be colonized with PNSP were young (≤20-years) and more frequently ate meat and dairy products, particularly pork (adjusted OR 52.30 [95% CI 8.72–313.60]) and milk derivatives (aOR 12.48 [4.01–38.82]). Consumption of fermented food was a protective factor (aOR, 0.02 [<0.01–0.13)].

**Interpretation:**

The prevalence of PNSP was high, but not associated with individual antibiotic use. Community-level interventions to reduce antibiotic consumption are urgently needed, as well as further investigations on antibiotic residues in food products to assess their role in the emergence and prevalence of PNSP.

**Funding:**

This work was supported by Oxford University Clinical Research Unit internal grants from the 10.13039/100010269Wellcome Trust Africa Asia Programme core grants (2015-2022—106680/Z/14/Z and 2022-2029—2022-2029—225167/Z/22/Z).

**Research in context**

**Evidence before this study**

A comprehensive search of scholarly journals from Pubmed/Medline was conducted. Peer-reviewed publications were included and publications prior 2000s were excluded, unless more recent evidence was lacking. Additional inclusion criteria were: participants (with focus on age group, occupation and sample size), geographic location of the study and settings (e.g., hospital, community). Initial search terms included: global + prevalence + *Streptococcus pneumoniae*, Review + *S. pneumoniae*, WHO + *S. pneumoniae*, children + *S. pneumoniae*, *S. pneumoniae* + infection + community, *S. pneumoniae* in children + Vietnam, VINARES project, Antimicrobial/Antibiotic/β-Lactam Resistance + *S. pneumoniae* + Vietnam, Penicillin Resistance/Penicillin Non-Susceptibility + *S. pneumoniae*, Non-susceptible *S. pneumoniae* + Vietnam.

The literature review highlighted the lack of information on the prevalence of PNSP in Northern Vietnam, particularly at the community level, and the need for further research to understand the burden and determinants of antibiotic-resistant strains in this region.

**Added value of this study**

Our study confirms high levels or penicillin non-susceptible *S. pneumoniae* in Vietnamese communities, and identifies specific factors associated with higher likelihood of colonisation. Individual antibiotic use was not associated with higher likelihood of colonisation, but consumption of animal food products was.

**Implications of all the available evidence**

While interventions to reduce the high levels of antibiotic use in communities and in livestock production are urgently needed, further research is also required to understand the mechanisms behind the observed effects in this study. In particular, we need to understand possible sources of occult exposure to antibiotics through residues in animal food products.

## Introduction

Lower respiratory tract infections caused by *S. pneumoniae* (pneumococcus) are a leading cause of morbidity and mortality worldwide, responsible for an estimated 1,189,937 deaths in 2016, the great majority of these occurring in low- and middle-income countries (LMICs).^1^ Beta-lactam antibiotics such as penicillin remain the preferred treatment for pneumococcal infections, though the global prevalence of penicillin-resistant/non-susceptible *S. pneumoniae* strains is increasing. Beta-lactam resistance is rarely associated with treatment failure but represents a growing threat in Asia, particularly in Vietnam, which is among the countries that face the highest levels of antimicrobial resistance (AMR) in the world.^2^ Several epidemiological studies have reported the prevalence of pneumococci with resistance or non-susceptibility to multiple antimicrobial agents, including penicillin and ceftriaxone, among the Vietnamese population at both clinical and community level.^3^^,^^4^ In a recent longitudinal study conducted on 221 children colonized with *S. pneumoniae* from the rural BaVi District, northern Vietnam, prevalence of multi-drug resistance (i.e., resistant to at least three antibiotics) increased from 31% in 1999, to 60% in 2007, and reached 80% in 2014. Of the *S. pneumoniae* isolates investigated in 2014, 78% showed intermediate resistance against penicillin (MIC >0.06 and ≤2 mg/L) and 12% were fully resistant (MIC>2 mg/L).^5^

In 2012–2013, the VINARES network reported a lower prevalence of 33% penicillin-resistant *S. pneumoniae* in routine clinical diagnostic specimens from 16 national and provincial hospitals across Vietnam, but this population also included adults.^6^ Similar resistance percentages were reported by the VINARES network in 2016–2017 (37%).^7^ These data emphasize the importance of identifying local key factors influencing antibiotic exposure to understand antibiotic resistance patterns at community level. The direct use of antibiotics is associated with higher prevalence of antibiotic-resistant bacteria, indeed limiting the use of antibiotics in primary care could reduce the overall levels of AMR.^8^ Furthermore, the use of antibiotics in livestock farming and aquaculture to treat and prevent infections and promote animal growth, could play an important role in AMR development and pose a risk to human health.^9^^,^^10^

To date, few rigorously evaluated, population-level interventions aiming to tackle AMR have been implemented and tested in Vietnam. The goal of this household survey was to provide behavioural and biological prevalence estimates to inform the design of a large population-based One Health trial, targeting inappropriate antibiotic use in a community from North Vietnam. We determined the prevalence of penicillin non-susceptible pneumococci (PNSP), and evaluated the quantitative roles of different factors that contributed to penicillin non-susceptibility in individuals carrying *S. pneumoniae*. The baseline measures of primary endpoints provided here will inform the design of interventions to reduce antibiotic intake and PNSP carriage prevalence.

## Methods

### Study population, data and sample collection

Between the 16th of July 2018 and the 10th of April 2019, a total of 1868 individuals from 389 households were randomly selected from a list of registered households provided by the local health authority for 19 villages in one commune of Binh Luc District, Ha Nam province, northern Vietnam.^11^ At the time of study design, this commune had a population of 9746 inhabitants distributed over 2638 households. All residents of selected households were invited to participate. No further eligibility criteria were applied. Quantitative survey data were collected from the primary caregiver of each household (Supplementary Fig. S1). Trained interviewers used a structured questionnaire in Vietnamese to collect comprehensive information about recent illness, healthcare seeking and antibiotic use. Complete paediatric history was recorded for children under the age of five including vaccination coverage, breastfeeding, and childcare attendance. Further data on hygiene practices and dietary habits, contact with animals and general socio-demographic characteristics were collected at household level. As part of a study to compare the reliability of different methods of swabbing, two-thirds of enrolled households were asked to provide a self-administered nasal swab to be collected on the next day after visit.^11^ The remaining one-third were asked to provide a nasopharyngeal swab administered by a local, trained health-worker. Swabs were collected in M40 Transystem Amies Agar Gel with charcoal, and transported to the laboratory in a cool box (2–8 °C) within 24 h from collection.

Households with children under-five were oversampled to ensure sufficient cases for this sub-group. Data were weighted based on total number of households in the commune (N = 2638) and by classifying households in “with” or “without” at least one child under-five. Survey weights were computed in RStudio (*srvyr* and *survey* package) to correct for differences between population frequencies observed in the sample and the actual population distribution. The *svydesign* function was used to create an object which combined the data frame and the survey design information needed for the analysis by survey modelling (*svyglm*) and summary.

### Ethics approval and consent to participate

The study was approved by Oxford Tropical Research Ethics Committee (Reference 552-17), the Institutional Review Board in Bio-medical Research of the National Institute of Hygiene & Epidemiology (Reference IRB-VN0105 7-01/2018) and the Ethics Committee of London School of Hygiene & Tropical Medicine (Reference 15831). Written informed consent was collected from all household members aged 18-years or older or parent/legal guardian of those under 18-years, and signed assent from those aged 12–18 years.

### *S. pneumoniae* isolation and identification

Nasal and nasopharyngeal swabs were transported to the laboratory of the National Institute of Hygiene and Epidemiology (NIHE) in Hanoi and inoculated on blood agar plates (BAP) supplemented with 5 μg of gentamicin. After overnight incubation, plates were carefully examined for pneumococcal colonies, typically surrounded by a greenish zone of alpha-hemolysis. Suspected pneumococcal colonies were streaked on BAPs and incubated overnight with an optochin disc in 5% CO_2_ atmosphere at 35–37 °C. Mass spectrometry with MALDI Biotyper v.3.0 (Bruker Daltonics, MA, USA) was performed on optochin-susceptible colonies to confirm *S. pneumoniae* identification. *S. pneumoniae* strains were stored at −80 °C in skim milk tryptone glucose glycerol medium and later retrieved for antimicrobial susceptibility testing.

### Antimicrobial susceptibility testing

*S. pneumoniae* colonies were suspended in sterile saline solution (0.85% NaCl) and turbidity was adjusted to 0.5 McFarland standard. Sterile cotton tips were used to evenly streak each suspension onto the surface of Müller-Hinton agar plates supplemented with 5% sheep blood (MHB) and the appropriate antimicrobial discs were applied on the MHB agar. The antibiotic susceptibility discs (Oxoid, Thermofisher Scientific, UK) used for antimicrobial susceptibility testing included: levofloxacin (5 μg), clindamycin (2 μg), trimethoprim-sulfamethoxazole (1.25/23.75 μg), tetracycline (30 μg), and erythromycin (15 μg). Plates were incubated overnight at 37 °C in 5% CO_2_ atmosphere. The radius of the zone of inhibition for each disc was interpreted according to 2020 Clinical Laboratory Standard Institute (CLSI) guideline.^12^ *S. pneumoniae* reference strain ATCC 49619 was used as a control.

Gradient test strips (Oxoid, Thermofisher Scientific, UK) were used to determine minimum inhibitory concentration (MIC) of *S. pneumoniae* to penicillin. Penicillin non-susceptible pneumococci (PNSP) were defined as having a MIC ≥0.12 μg/ml, which corresponds to CLSI breakpoint for both oral penicillin and parenteral penicillin in meningitis cases, and to EUCAST 2020 breakpoint.^12^^,^^13^ Penicillin resistance was defined according to CLSI breakpoints for oral penicillin, i.e., MIC ≥ 2 μg/ml.^12^^,^^13^ Resistance to 3 or more classes of antibiotics was referred to as multi-drug resistance (MDR).^14^

### Data transformation

**Fig. 1:**
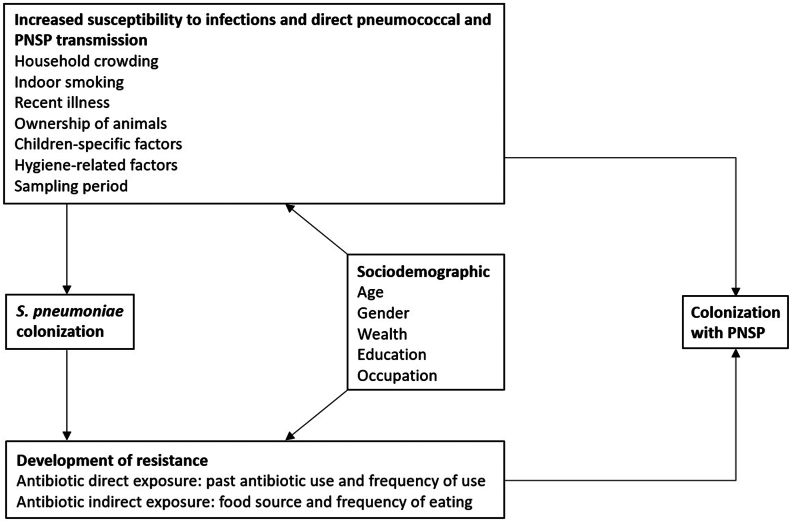
Diagram of the risk factors investigated as putative predictors for carriage of *S. pneumoniae* isolates non-susceptible to penicillin (PNSP).

Microsoft Excel 2020 and R v.4.1 were used for data curation and analysis. Risk factors for PNSP carriage were investigated according to the diagram in Fig. 1 and are data curation details are summarised in Supplementary Table S2.

A wealth index was generated by principal components analysis (PCA) as a measure of the socio-economic status of the interviewed households. This index was constructed from household features and selected assets (floor, roof, and wall materials, crowding (number of people per sleeping room), ownership of electricity, radio, television, telephone, mobile phone, refrigerator, bed, table and chair set, sofa, computer, tablet or iPad, fan, air conditioner, gas cooker, electric cooker, washing machine, bicycle, motorcycle, tractor, car or truck, ship or boat). All variables were converted to binary variables, except for household crowding. We did not standardise our data, so we used the correlation matrix to ensure that all data had equal weight.^15^

Factors that could facilitate *S. pneumoniae* colonization, promote direct or indirect intake of antibiotics leading to resistance among colonising *S. pneumoniae*, or factors associated with direct PNSP colonization were investigated. Household overcrowding was defined according to WHO guidelines as more than three persons per room.^16^ The sampling period was investigated due to seasonality in pneumococcal transmission and antibiotic use.^17^ Hygiene-related factors included drinking water source and treatment, use of manure, toilet facility, and handwashing practices. Factors specific for children included hospitalization after birth, vaccination with pneumococcal conjugate vaccine (PCV), childcare attendance and breastfeeding practices, among others.

Antibiotic consumption was examined based on the latest use and the frequency of use in the past 6 months. Indirect exposure to antibiotic residues in food was evaluated by investigating the type of meat eaten and the frequency of consumption per week. Probiotic intake via fermented products was investigated for its potential protective effect.

### Statistical analysis

The total sample size of 1502 for the survey was used to compare recovery of *S. pneumoniae* in different swabbing groups, and has been described elsewhere.^11^ Records with missing data for exposure variables were retained where possible by assigning them to an “unknown” category, as detailed in Supplementary Table S5. In this study, we assumed that 11% of the whole population would carry *S. Pneumoniae*, based on a study in Vietnam that used a similar ratio of oversampling households with children under 5-years old (unpublished PhD). From 1500 samples, we expected to recover *S. Pneumoniae* from 165, which would give a margin of error of between 4.68% and 4.9% for prevalence estimates, using a prevalence of PNSP from previous estimates of 90%, and a 95% confidence level for prevalence estimates, and a design effect between 1.03 and 1.15 (based on intracluster correlation coefficient (ICC) in the range of 0.05–0.30, and an average of 1.5 samples per household from 112 households).

Statistical analyses were adjusted for survey design using the R svyr package (standard errors adjusted for correlation within households and sampling weights applied). Univariable and multivariable logistic regression models were used to explore association between individual and household factors associated with PNSP carriage. Multivariable logistic regression models were run for each explanatory variable, adjusting for age and wealth status. Mediation analysis was conducted to assess whether the observed associations might be mediated by differences in individual antibiotic use,^15^ thus models were further adjusted for antibiotic use in the past four weeks (Model 3, Table 1 and Table 2).

**Table 1:** Univariable and multivariable logistic regression to determine association between individual and household risk factors for PNSP carriage.

Variable	Categories	N^a^	PNSP (n)	PNSP (%)	Crude OR [95% CI]	p-value	Adjusted OR [95% CI], Model 2	p-value	Adjusted OR [95% CI], Model 3	p-value
**Sociodemographic**										
Age (years)	≥20	47	24	51.1	ref					
	5–19	33	31	93.9	11.89 [2.33–60.63]	0.003				
	<5	48	42	87.5	7.23 [2.53–20.71]	<0.001				
Wealth	low	44	33	75	ref					
	middle	42	32	76.2	0.92 [0.33–2.62]	0.883				
	high	42	32	76.2	0.98 [0.35–2.75]	0.97				
**Direct exposure to antibiotics**										
Last ABU	≥6 months ago	19	8	42.1	ref					
	2–5 months ago	23	14	60.9	1.17 [0.32–4.31]	0.816	0.37 [0.08–1.61]	0.187		
	last month	33	30	90.9	8.25 [1.67–40.79]	0.01	1.21 [0.12–12.07]	0.871		
	unknown	53	45	84.9	6.37 [1.86–21.89]	0.004	1.97 [0.51–7.58]	0.325		
ABU frequency (6 months)	not used	19	8	42.1	ref					
	1-2 times	23	17	73.9	2.25 [0.57–8.86]	0.249	0.54 [0.10–2.91]	0.474		
	≥3 times	31	27	87.1	7.25 [1.74–30.18]	0.007	1.07 [0.16–7.35]	0.943		
	unknown	55	45	81.8	4.25 [1.28–14.10]	0.02	1.36 [0.38–4.93]	0.64		
**Increased susceptibility to infection, pneumococcal and PNSP transmission**										
House crowding	no	18	13	72.2	ref					
	yes	109	83	76.1	0.74 [0.23–2.42]	0.625	0.43 [0.09–1.97]	0.681	0.41 [0.08–1.95]	0.258
	unknown	1	1	100						
Indoor smoking	no	88	66	75.0	ref					
	yes	39	30	76.9	1.31 [0.51–3.41]	0.571	1.21 [0.40–3.64]	0.478	1.20 [0.39–3.66]	0.751
	unknown	1	1	100						
Drinking water	rain water only	49	33	67.3	ref					
	other source	79	64	81.0	2.16 [0.91–5.12]	0.082	1.59 [0.59–4.32]	0.361	1.65 [0.59–4.56]	0.335
Illness in the last 2-weeks	no and unknown	103	74	66.1	ref					
	yes	25	23	92.0	3.24 [0.66–15.89]	0.151	1.60 [0.19–13.16]	0.664	1.64 [0.30–8.89]	0.561

Regressions were adjusted for age and wealth (Model 2) and for age, wealth and antibiotic use (Model 3).

a All analyses included 128 participants colonized with *S. pneumoniae*.

**Table 2:** Univariable and multivariable logistic regression to determine association between diet and risk for PNSP carriage.

Variable	Categories	N^a^	PNSP (n)	PNSP (%)	Crude OR [95% CI]	p-value	Adjusted OR [95% CI], Model 2	p-value	Adjusted OR [95% CI], Model 3	p-value
**Indirect exposure to antibiotics. Frequency of eating:**										
Chicken	<once per week	76	51	67.1	ref					
	=once per week	13	9	69.2	0.83 [0.22–3.22]	0.79	0.94 [0.25–3.48]	0.923	0.94 [0.25–3.48]	0.924
	>once per week	39	37	94.9	9.75 [2.10–45.31]	0.004	7.60 [1.22–47.038]	0.03	7.58 [1.32–43.75]	0.027
Pork	<once per week	23	7	30.4	ref					
	=once per week	30	23	76.7	9.15 [2.43–34.39]	0.001	13.50 [2.74–66.61]	0.002	13.06 [2.50–68.31]	0.003
	>once per week	75	67	89.3	28.90 [8.51–98.08]	<0.001	53.05 [9.10–309.33]	<0.001	52.30 [8.72–313.60]	<0.001
Beef	<once per week	85	58	68.2	ref					
	=once per week	12	10	83.3	1.38 [0.27–7.05]	0.949	1.45 [0.33–6.27]	0.618	1.46 [0.33–6.28]	0.615
	>once per week	31	29	93.5	6.46 [1.39–30.13]	0.018	3.93 [0.55–27.87]	0.169	3.84 [0.58–25.65]	0.163
Milk products	<once per week	31	10	32.3	ref					
	=once per week	7	6	85.7	12.50 [1.26–123.80]	0.031	4.69 [0.40–55.67]	0.208	5.25 [0.36–77.20]	0.224
	>once per week	83	74	89.2	18.12 [6.12–53.65]	<0.001	12.08 [3.89–37.48]	<0.001	12.48 [4.01–38.82]	<0.001
Fermented food	<once per week	61	51	83.6	ref					
	=once per week	20	2	10.0	0.03 [0.01–0.14]	<0.001	0.02 [<0.01–0.14]	<0.001	0.02 [<0.01–0.13]	<0.001
	>once per week	43	40	93.0	2.72 [0.63–11.64]	0.177	2.40 [0.47–12.31]	0.293	2.45 [0.64–9.38]	0.19
Tofu	<once per week	15	14	93.0	ref					
	=once per week	22	11	50.0	0.08 [0.01–0.73]	0.025	0.06 [<0.01–1.18]	0.064	0.06 [<0.01–1.29]	0.072
	>once per week	84	65	77.0	0.24 [0.03–2.03]	0.19	0.26 [0.01–4.52]	0.349	0.29 [0.02–4.67]	0.381

Regressions were adjusted for age and wealth (Model 2) and for age, wealth, and antibiotic use in the past 4 weeks (Model 3).

a All analyses included 128 participants colonized with *S. pneumoniae*, except fermented food where all models had 124 participants and milk products and tofu where all models had 121.

### Role of the funding source

The funders had no role in study design, data collection, data analysis, interpretation, or writing of the report.

## Results

### Study population

A total of 1502 individuals from 324 households, agreed to participate in the study (80.4% population response rate). Of these, no samples were collected from four participants and they were excluded from the analyses. No data were missing for other variables included in models. Demographic and socio-economic characteristics of the 1498 study participants are summarized in Supplementary Table S3. The median age was 29 years (interquartile range 8–49), with 48.2% (722) males and 51.8% (776) females. Individuals with high (510), middle (489) and low (499) socio-economic status represented 34%, 32.7% and 33.3% of the study population, respectively. Data on education and occupation were collected from 968 participants. The great majority of adults did not receive any education in the past (79.2%, 767).

A total of 1498 swabs were collected, including 1002 self-administered nasal swabs and 496 nasopharyngeal swabs administered by health workers (HW) (Supplementary Table S2). Four samples were lost and 345 samples were discarded due to faulty transport medium, leaving 1149 samples for testing. Of these, *S. pneumoniae* was successfully cultured from 10.3% (83/799) of self-administered swabs and 14% (49/350) of HW-administered swabs, and therefore recovered from 132 individuals in total. Children under-five (N = 242) were more colonized than other age categories, representing 35.6% (47/132) of total *S. pneumoniae* carriers. As a consequence, the majority of *S. pneumoniae* isolates (59.1%, 78/132) were detected in participants still attending school. Wealth terciles shared a similar proportion of *S. pneumoniae* carriers (34.1%, 33.3% and 32.6%, from high to poor socioeconomic status, respectively).

### Antimicrobial susceptibility testing

**Fig. 2:**
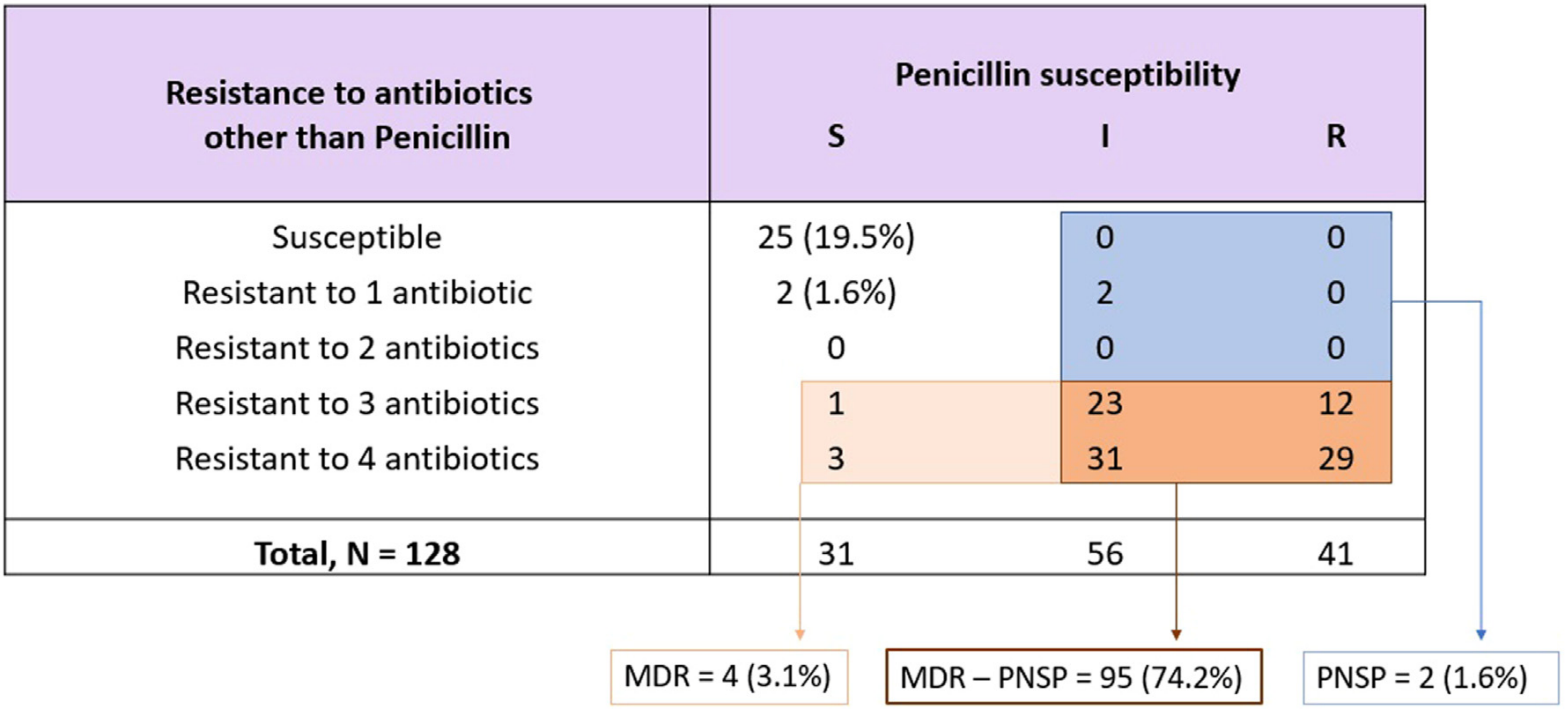
Antibiotic susceptibility of *S. pneumoniae* isolates among the population. MDR = multi-drug resistance; PSSP = penicillin susceptible *S. pneumoniae*; PNSP = penicillin non-susceptible *S. pneumoniae*.

Data on *S. pneumoniae* antibiotic susceptibility were obtained for 97% (128/132) of isolates. Antimicrobial susceptibility testing (AST) results are summarized in Fig. 2 and given in more details in Supplementary Table S4. Briefly, 19.5% of *S. pneumoniae* isolates were susceptible to all antibiotics tested while the vast majority (77.3%, 99/128) of isolates were found with MDR, of which 96.0% (95/99) were non-susceptible to penicillin (PNSP). In total, 75.8% of all isolates were PNSP (97/128). There was no resistance to levofloxacin; the proportion of *S. pneumoniae* resistant to trimethoprim-sulfamethoxazole and tetracycline were 63.3% and 66.4%, respectively; the proportion of resistance to clindamycin and erythromycin were 77.3%.

### Risk factors for PNSP carriage in colonized population

Risk factors associated with the carriage of PNSP versus penicillin susceptible *S. pneumoniae* (PSSP) were investigated in individuals colonised with pneumococcal isolates from whom AST data were successfully obtained. Key results for logistic regression analysis are shown in Table 1, Table 2. Crude odds ratio of additional determinants explored for the study are provided in the Supplementary Table S5.

There was a positive association between age and prevalence of PNSP, with children under-five and participants aged 5–19 years more likely to be colonized than older individuals (under-five years: crude OR, 7.23 [95% CI 2.53–20.71]; 5–19 years: cOR, 11.89 [2.33–60.63]; Table 1). Similarly, individuals attending school had higher PNSP colonization proportions than workers and unemployed participants (cOR, 5.96 [1.85–19.18]; Supplementary Table S5).

**Fig. 3:**
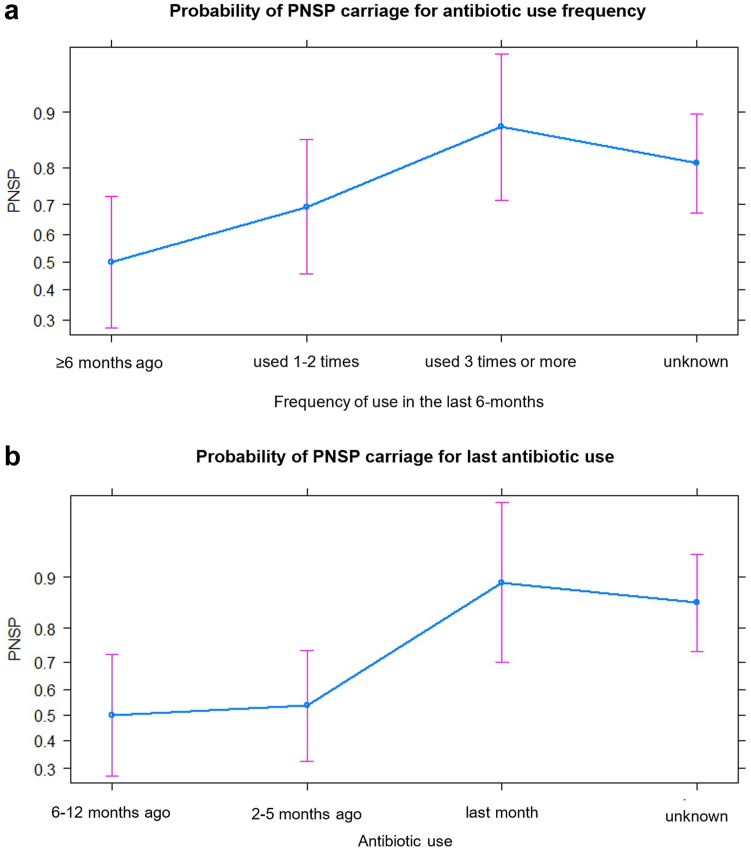
Plot in linear predictor (logit) scale of PNSP carriage by antibiotic recent use and by frequency of use in the past 6 months. The tick marks on the vertical axis are labelled in mean (P, probability) scale with a 95% CI (pink bar).

PNSP carriage was significantly higher in participants who used antibiotics in the month before collection (cOR, 8.25 [1.67–40.79]) and those who consumed antibiotics three times or more in the past 6 months (cOR, 7.25 [1.74–30.18]). The progressive increase of PNSP prevalence against the last antibiotic use (ABU) and ABU frequency is shown in Fig. 3. Out of 53 Individuals who did not know whether they had consumed antibiotics within the previous 6-months, 45 (84.9%) were colonized with PNSP, a similar colonization rate to individuals reporting using antibiotics at least once in the last month (90.6%, 30/33). However, after adjusting for age and wealth, the effects of recent or frequent antibiotic exposure were no longer seen. None of the factors related to susceptibility to infection or pneumococcal and PNSP transmission were associated with PNSP carriage, including history of illness in the past 2 weeks (aOR, 1.60 [0.19–13.16]).

Due to the small sample size, risk factors specific for children under-five were not statistically investigated. Data on day-care attendance were available for 48 children of whom 34 colonized by PNSP (81.2%). None of the children was vaccinated with PCV. PNSP carriage was detected in 40 of the 44 children (90.9%) who were not exclusively breastfed in the first 6 months of life, compared to 2 of 4 (50%) who were exclusively breastfed, and in almost all children who were hospitalized after birth (95.6%, 22/23).

A higher frequency of PNSP colonization was observed in individuals sampled in autumn and winter compared to spring and summer (cOR, 11.41 [2.33–55.78], Supplementary Table S5) particularly in November–December compared to March–April (cOR 14.38 [2.89–71.55]). However, this association was not considered to represent a seasonal effect, as a high number of susceptible isolates were collected at one single location over 3 days in March and April.

To assess the potential for food consumption to lead to indirect antibiotic exposure and development of PNSP carriage, we investigated the frequency of consumption of chicken, beef, pork, and milk products per week for each household. We also explored the possible protective effect of probiotics in fermented food. Four individuals colonised with *S. pneumoniae* lacked data regarding fermented food consumption, and seven lacked data regarding consumption of milk products and tofu. For these variables, model 1, 2 and 3 analyses were conducted with 124 and 121 individuals respectively, instead of the total 128. Frequent consumption was strongly associated with increased proportions of PNSP colonization for all types of meat and dairy products investigated (Table 2). In particular, the probability of PNSP carriage increased with increasing frequency of eating pork (once per week: aOR, 13.50 [2.74–66.61]; more than once per week: aOR, 53.05 [9.10–309.33]) and milk products (once per week: aOR, 4.69 [0.40–55.67]; more than once per week: aOR, 12.08 [3.89–37.48]). On the other hand, consuming fermented food at least once per week significantly reduced probability of colonization (once per week: aOR, 0.02 [<0.01–0.12]).

After adjusting for possible differences in antibiotic use between participants, the risk of PNSP carriage for those frequently eating pork remained almost unvaried (Model 3: aOR, 52.30 [8.72–331.60]), while the odds ratio slightly increased for milk products consumption (aOR, 12.48 [4.01–38.82]). The protective effect associated of a moderate consumption of fermented food remained unchanged (aOR, 0.02 [<0.01–0.13)].

## Discussion

In this population-based study, we report prevalence estimates of penicillin non-susceptible *S. pneumoniae* (PNSP) carriage and explored sociodemographic, behavioural and environmental factors potentially associated with PNSP colonisation in a rural community in northern Vietnam. The high proportion of healthy individuals carrying multidrug-resistant pneumococci (77.3%, 99/128) among carriers of pneumococci observed in the study, most of which were with reduced susceptibility to penicillin (75.8%, 97/128), indicates how consistently resistant strains are circulating in this community. Among pneumococci carriers, colonisation with PNSP was dependent on age with a prevalence of 87.5% in children younger than 5-years, of 93.9% in adolescents aged 5–19 years, and of 51.1% in adults aged 20- years or older. This is not surprising as children are considered the most important vector for the horizontal spread of *S. pneumoniae* in communities, especially of antibiotic-resistant strains.^18^ Pneumococci can be asymptomatically carried for longer in children, increasing the probability of antibiotic pressure on the bacteria.^19^ In Vietnam, high levels of non-susceptibility to beta-lactam antibiotics among pneumococci have been previously reported in both hospitalized and asymptomatic children.^20^

We did not find evidence for an association with PNSP carriage for risk factors that increased susceptibility to infection or transmission of pneumococci or PNSP. Environmental factors such as crowding and smoking, can also influence the composition of the nasopharyngeal niche affecting the overall prevalence of pneumococci, and potentially of PNSP.^21^ However, no significant associations between these factors and colonisation with PNSP was observed.

Consumption of pork was the strongest risk factor for PNSP colonization, alongside consumption of other meat and dairy products. The rapid economic growth of Vietnam over the last decades has greatly increased local meat production levels. In 2012, pork already represented 80% of the total meat produced in Vietnam.^22^ In spite of the introduction of the Animal Husbandry Law banning the use of antimicrobial growth promoters (AGPs) in commercial feeds in 2018,^23^ livestock are still often fed with industrial feed enriched with AGPs. Two Vietnamese surveys suggested that antimicrobials used in animal feed represent between 15 and 20% of total antimicrobial usage in chicken farming and the in-feed antimicrobial consumption in pigs is commonly greater than in chicken production.^9^^,^^24^ The consumption of meat therefore poses a potential danger for human health as antimicrobial residues in food may facilitate the selection of resistant bacteria. In a recent study performed in Hong Kong,^25^ pre-school children were shown to be exposed to low-level veterinary antibiotic residues in food on a daily basis. Penicillin had the highest concentration among the antibiotics investigated, and the highest detection rates in food and participants’ urine samples. The absorption of antibiotics from food sources and their excretion in urine can influence the gut microbiota but also other commensal flora, including the nasopharynx. This might be the reason why high levels of PNSP carriage where markedly correlated to frequent meat consumption. The source of meat products might also be of relevance since the compliance of market sellers, mostly smallholders, with AGP regulations is harder to assess than for large-scale farmers. However, the lack of information on household’s meat source from this study makes it difficult to draw conclusions.

Finally, the direct administration of antibiotics in animals for growth promotion and for routine prevention of diseases, could also result in final meat products containing antibiotic residues. We investigated the use of antibiotics in household’s farm animals in the event that these could be used as food source for the family unit rather than for selling but no significant association between antibiotic use and PNSP carriage was observed.

The consumption of moderate quantities of fermented products had a significant protective effect against PNSP colonization. Fermented food is rich in probiotics and in Vietnam is often used as condiments or within the main meal itself.^26^ Probiotics can have positive effects on the health of the host, reducing susceptibility towards pathogenic infections by shaping microbial community composition. For instance, *Corynebacterium spp.* are typical commensals of the nasal human mucosa and important probiotics of fermented food such as fish sauce.^27^ These bacteria tend to be overrepresented in children free of *S. pneumoniae*; *Corynebacterium accolens* is thought to release antibacterial free fatty acids to inhibit *S. pneumoniae* growth^28^ and *Corynebacterium pseudodiphtheriticum* plays a role in the enhancement of respiratory immune responses against *S. pneumoniae* infections in mice.^29^

In Vietnam, the type of food consumed changes throughout the year and varies according to regional, cultural, ethnic, and agricultural practices,^26^ so seasonal patterns and consumption of food products may correlate. We observed a possible seasonal pattern of PNSP, which could be mediated by seasonal changes in diet or seasonal differences in pneumococcal carriage and antibiotic use, as previously reported.^30^ Further investigations related to seasonality and patterns of antibiotic and food consumption should be conducted.

The high frequency of PNSP colonisation reported here is alarming since it is easy for healthy individuals to directly acquire resistant isolates once these become predominant at community level, regardless of their individual use of antibiotics.^4^ Resistance in carriage isolates can lead to the emergence of resistance in clinical isolates with serious consequences on current treatment regimens for pneumococcal pneumonia. The PNSP isolates in the current study showed additional resistance to erythromycin and trimethoprim-sulfamethoxazole, two recommended oral alternatives for treating acute respiratory infections. Other injectable alternatives such as ceftriaxone, chloramphenicol and vancomycin are not convenient for use in primary care and have numerous side effects.^31^ If the susceptibility profile of pneumococci that we observed in the present study is confirmed in clinical isolates, and if the non-susceptibility to penicillins escalates to resistance, we would be left with limited therapeutic options against *S. pneumoniae*, e.g., oral third-generation cephalosporins and quinolones, that belong to the Watch category in the WHO AWaRe classification.

Our study was limited by the small sample of participants from whom *S. pneumoniae* were recovered. Recovery was slightly lower for self-administered compared to HW-administered swabs (10.3% and 14.0% respectively), but the low carriage was mainly because our samples were taken from the general population, including adults. We have discussed determinants of carriage previously,^11^ but the recovery rate was similar to another general population sample in Vietnam, where 11% of healthy children and adults carried *S. pneumoniae*.^32^ In our study, 35.6% of children under 5-years carried *Streptococcus pneumonia*, which compares with other community-based studies among children under 5-years in Vietnam where carriage ranged from 28.7 to 52%, depending on the age composition of the participants, sample method, time period, and location.^4^^,^^5^^,^^33^ The small sample size affected the power of statistical tests, as we were unable to conduct sub-group analyses of specific risk factors for PNSP carriage in children under-five, where *S. pneumoniae* is predominantly found. We used disc diffusion rather than broth microdilution because it was simpler and more cost-effective, and has been used in similar population-based studies in Vietnam and elsewhere.^5^^,^^34^^,^^35^ We are unable to report on serotype distribution, specific resistance mechanisms, or clonal complexes to characterise *S. Pneumoniae* in this population. We did not perform serotyping or whole genome sequencing (WGS), as our objective was to evaluate the prevalence of PNSP carriage and identify behavioural and environmental determinants. Our results may not be generalizable to urban areas or other regions of Vietnam. Moreover, it was not possible to collect comprehensive data on all factors that can influence the commensal flora, which limited our ability to draw conclusions about the role of commensal flora in protecting against colonisation with PNSP. Finally, measurement of risk factors was based on recall, which may be subject to recall biases. Recollection of antibiotic use was particularly problematic, and 41% of participants could not state whether they had used antibiotics and 43% could not say how frequently they had used antibiotics in the last 6 months. This misclassification is unlikely to have differed by PNSP status, so would have biased the associations towards the null. However, we were still able to detect associations between recent and frequent antibiotic use and PNSP, suggesting that without misclassification bias, these effects may have been even stronger.

The significant association observed between consumption of animal products and PNSP carriage requires further investigation. The quantification of antibiotic residues in meat samples in future studies would help to better understand the role of meat consumption in PNSP carriage.

Our study demonstrated a high prevalence of AMR among commensal *S. pneumoniae* in a Vietnamese rural community, in particular non-susceptibility to beta-lactams. The association between nasopharyngeal carriage of PNSP and antibiotic intake did not persist in adjusted logistic regression models. In contrast, the consumption of chicken, pork, and dairy products remained constantly and strongly associated with PNSP carriage. The consumption of fermented food and tofu had a small and inconsistent protective effect against colonisation with PNSP that remains to be confirmed. These results underline the importance not only of enforcing the ban on sales of antimicrobials over the counter for human health, but also to rationalize the antibiotic use in the farming industry. Population-based interventions aimed at reducing consumption of antibiotics in the community are therefore strongly recommended in an attempt to moderate the levels and impact of inappropriate use and the prevalence of resistant/non-susceptible pneumococci in the general population. Further research is needed to better understand the relationship between diet, and in particular meat consumption, and PNSP.

## Contributors

SL, HRvD, BNTV, HATN, HHT conceptualised and designed the study, acquired funding, and had final responsibility for the decision to submit for publication. TCTN coordinated study implementation and planned and executed research activities with support from TDT and DTN. BNTV, HHT, HATN, TDP, QDP, KHTT, THTN, and TTN conducted lab analyses. CT, TST, DTVV, and MvW led data curation and managed and prepared survey datasets and verified the data. CT conceptualized and conducted analyses and wrote the first draft of the manuscript. TK supervised analysis and interpretation. MvW, TK, CT, TST, DTVV and SL had access to the raw data. DAD supervised implementation and was responsible for liaison with local partners. All authors reviewed and commented on the manuscript and read and approved the final version.

## Data sharing statement

Datasets generated and analysed during the current study are not publicly available as this was not included in the consent process, but anonymised datasets can be made available from the corresponding author on reasonable request.

## Declaration of interests

H. Rogier van Doorn is a board member for Wellcome SEDRIC (Surveillance and Epidemiology of Drug Resistant Infections Consortium). Thomas Kesteman received support from Medecins Sans Frontieres France (MSF-OCP) to attend a conference, is member of the scientific committee for the Mini-Lab project on AMR, MSF-OCP 2016–2022. Max van Wijk received consulting fees from the Quadripartite Joint Secretariat on AMR to draft the first biannual report on AMR in 2022, and from IS Global to contribute to a report on AMR in 2022/23, commissioned by DG SANTE/European Commission. The remaining authors declare that they have no competing interests.

## Appendix A Supplementary data

Supplementary Fig. S1 and Tables S2–S5

Tacoli_PNSP_Abtract_VNese final
