# Supplementary material for: Prevalence and determinants of nasal carriage of penicillin non-susceptible Streptococcus pneumoniae: a cross-sectional household survey in northern Vietnam
Source: Lancet Reg Health West Pac. 2025 Jan 8;54:101282. doi: 10.1016/j.lanwpc.2024.101282 (PMC11764309; doi:10.1016/j.lanwpc.2024.101282)
Supplement: Supplementary Fig. S1 and Tables S2–S5 [file mmc1.docx]

# **Supplementary Tables and Figures**

## **Figure 1S**. Flow diagram of the household survey, detailing participant recruitment, swab collection, and laboratory results for *S. pneumoniae* isolates that were successfully retrieved after storage and tested for antimicrobial susceptibility (AST).


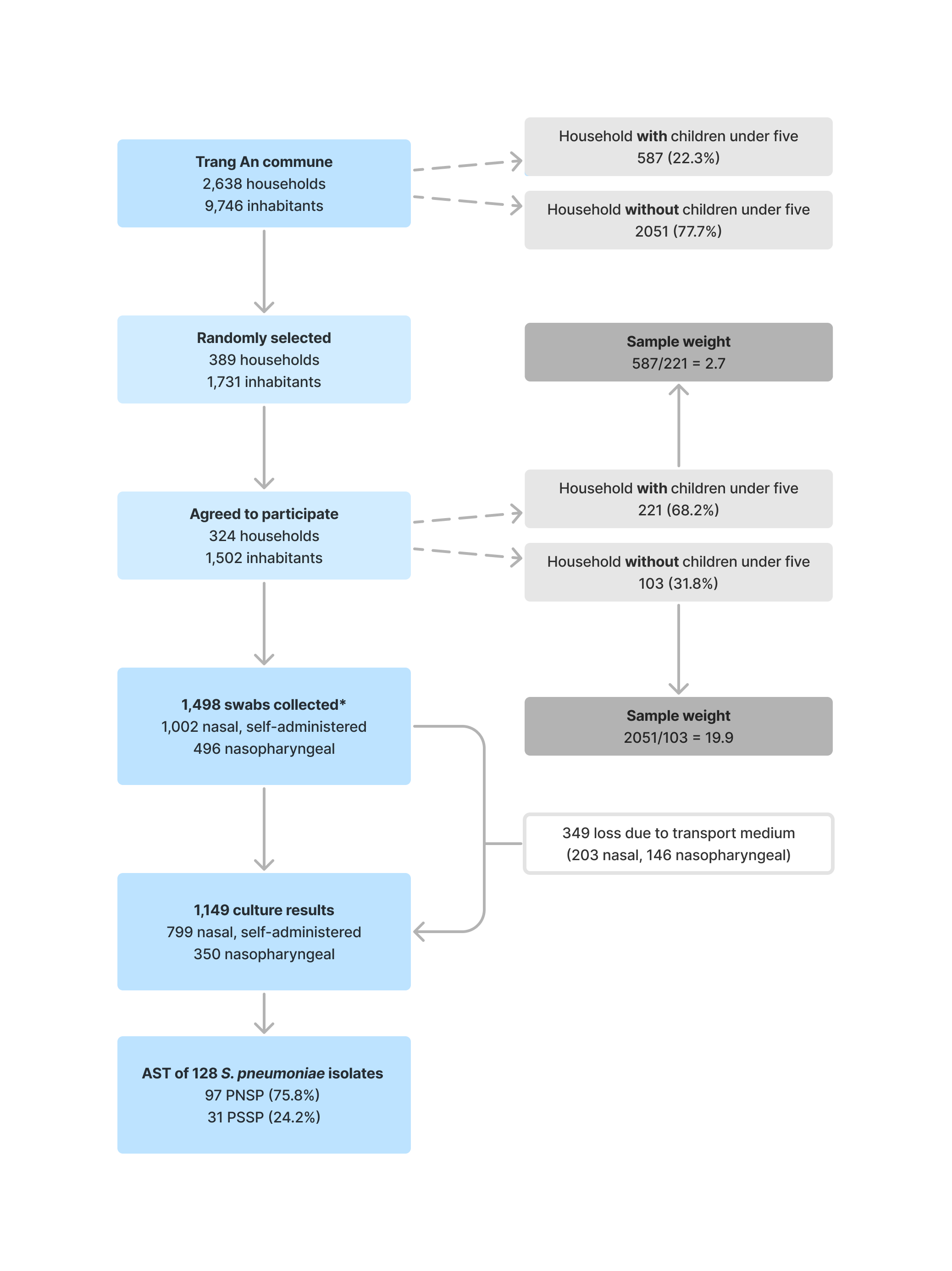


*Samples were not collected from four household members, resulting in 1,498 total participants.

**Table 2S**: List of curated variables

| **Variable** | **Levels** | **Description** |
| --- | --- | --- |
| Penicillin non-susceptible *Streptococcus pneumoniae* (PNSP) | No  Yes | Colonization with PNSP was defined confirmation of S. pneumoniae colonies by MALDI-TOF, and susceptibility was tested by disc diffusion. |
| Age | - | Age in years based on the difference between the date of the household interview (see section 1 of the survey; supplementary file 1) and the birth date (see question 2.2 of the survey; supplementary file 1). |
| Sex | Female  Male | Sex is based on the self-reported sex (see question 2.2 of the survey; supplementary file 1). Representatives of each household were asked to answer this question for all households. Variable was only analysed for individuals > 16 years-of-age. |
| Education | College/university  Professional school  Upper secondary  Lower secondary  Primary and lower  No education  Unknown | Educational level based on the self-reported, highest level of education (see question 2.5 of the survey; supplementary file 1). Representatives of each household were asked to answer this question for all households. Variable was only analysed for individuals > 16 years-of-age. |
| Occupation | Farmer  Labourer  Factory worker  Office worker  Shop/retail/hospitality  Other work  Student  No work/unknown | Occupational level based on the self-reported current occupation (see question 2.9 of the survey; supplementary file 1). Representatives of each household were asked to answer this question for all households. Variable was only analysed for individuals > 16 years-of-age. |
| Socio-economic status (SES) | Low income  Middle income  High income | Cumulative score of principal component analysis (PCA) of direct observations of general assets and house construction ((i.e.. flooring material, roofing material, wall material, crowding (number of people per sleeping room), ownership of electricity, radio, television, telephone, mobile phone, refrigerator, bed, table and chair set, sofa, computer, tablet or iPad, fan, air conditioner, gas cooker, electric cooker, washing machine, bicycle, motorcycle, tractor, car or truck, ship or boat; see section 3 of the survey; supplementary file 1). Factors that may have had a direct effect on colonization with resistant bacteria were excluded from the PCA (i.e., handwashing places, availability of soap, type of toilet, water source, ownership of animals). Scores were subdivided into three wealth terciles. |
| Illness ^A^ | No and unknown  Yes | “Yes” for participants that reported to suffer from diarrhoea, cough and/or fever in the two weeks prior or during the survey interview. “No and unknown” for all other (see section 5 of the survey; supplementary file 1) |
| Antibiotic use 4 weeks ^A^ | No and unknown  Yes | “Yes” for participants that reported using antibiotics 4 weeks before or during the survey interview. “No and unknown” for all other (see question 5.26 of the survey; supplementary file1). |
| Sampling period | Period 1 (Jul-Sep)  Period 2 (Nov-Dec  Period 3 (Mar-Apr) | Sampling throughout the study was subdivided into three distinct periods: (1) Period 1 between July and September 2018, (2) Period 2 between November and December 2018, and (3) Period 3 between March and April 2019. There was no sampling for >1 month between each sampling period. |
| Owning animals | No  Yes | “Yes” for participants that reported to have at least one animal (i.e., cattle, horses, donkeys, mules, goats, chicken, quails, pigs, ducks, geese, swans, fish, and/or pet animals) (see question 3.16 of the survey; supplementary file 1). |
| Antibiotic use in animals | No animals  Animals and antibiotic use  Animals and no antibiotic use | Based on “owning animals” and self-reported use of antibiotics in animals (see questions 3.16 and 3.17 of the survey; supplementary file 1). |
| Owning livestock | No livestock  1 species  2 species or more | Based on the “owning animals” variables with cattle, horses, donkeys, mules, goats, chicken, quails, pigs, ducks, geese, swans, and fish considered as livestock. |
| Beef consumption | Less than once per week  Once per week  More than once per week | Food consumption was based on the self-reported consumption of selected food type (see question 3.30 of the survey; supplementary file 1). Representatives of each household were asked to indicate how frequently they ate each subtype with the following 5 answer options: never, less than once per week, once per week, more than once per week, or every day. |
| Chicken consumption | Less than once per week  Once per week  More than once per week |  |
| Tofu consumption | Once per week and less  More than once per week |  |
| Fermented food consumption | Once per week and less  More than once per week |  |
| Drinking water source | Improved source  Rainwater | “Rainwater” for households that reported exclusive use of rainwater as a main drinking water source, “Improved” for households using any additional/other source (see question 3.19 of the survey; supplementary file 1). |
| Water Sanitation and Hygiene (WASH) | Worse  Middle  Better | The WASH condition was based on three factors: (1) using rainwater as a source of drinking water, (2) boiling water before consumption, (3) having a flushed toilet. “Better” were all households that met these three criteria, “middle” when one was missing, and “worse” for all other households. |
| Handwashing | Often  Not often | The handwashing behaviour was determined for several activities and scored as never (0 points), sometimes (1 point), most of the time (2 points), always (3 points). The mean score was calculated for 7 activities (i.e., after toilet, before cooking, after cooking, before eating, after eating, after handling animals and after sneezing or coughing). Mean scores equal and higher than 1.5 were considered as “often”. |
| Hospital exposure ^A^ | No  Yes | “Yes” for participants that reported to have been admitted, visited, or worked at a hospital within the last month before the survey interview. |
| Delivery | Natural delivery  Caesarean section | “Caesarean section” for participants that reported a child was delivered through caesarean section (see question 4.1 of the survey; supplementary file 1), “Natural delivery” for the remaining participants under the age of 5. |
| Hospitalisation | Less than 1 day  One day or more | Based on questions 4.3 and 4.4 (see supplementary file 1). |
| Early breastfed | Yes  No | “Yes” for children under 5 years-of-age who were breastfed within the first hour after delivery and “No” for all other children. This cut-off was based on WHO’s guidelines for early initiation of breastfeeding. |
| Exclusive breastfed 4 months | Yes  No | “Yes” for children under 5 years-of-age who were exclusively breastfed with milk until the age of 4 months, and “No” for all other children. |
| Liquids first 3 days | Only mother milk  Other milk / instant formula | “Yes” for children under 5 years-of-age who were exclusively breastfed with milk during the first 3 days, and “No” for all other children. |
| Fully vaccinated | Yes  No | As per schedule of the national expanded program on immunization (EPI) in Vietnam. Children were considered fully vaccinated when:   - Younger than 2 months: BCG + HepB - Between 2 and 3 months: above + 1 OPV + 1 pentavalent - Between 3 and 4 months: above + 1 OPV + 1 pentavalent - Between 4 and 9 months: above + 1 OPV + 1 pentavalent - Between 9 and 18 months: above + 1 MMR - Older than 18 months: above + 1 MMR   Fully vaccinated children (at the age of 18 months) hence received 1 BCG, 1 HepB, 3 OPV, 3 pentavalent and 2 MMR vaccines. |

## **Table 3S.** Socio-demographic and econmic characteristics of the study population (N=1,498), and of *S. pneumoniae* carriers (N=132).

| **Characteristics** | **Study population, n (%)** | ***S. pneumoniae* carriers, n (%)** |
| --- | --- | --- |
| **Total, N** | **1498** | **132** |
| **Age**  < 5 years  5-19 years  20-29 years  30-39 years  40-49 years  50-59 years  ≥ 60 years | 243 (16.2)  293 (19.5)  242 (16.2)  268 (17.9)  121 (8.1)  169 (11.3)  162 (10.8) | 50 (37.9)  35 (26.5)  6 (4.5)  21 (15.9)  2 (1.5)  6 (4.6)  12 (9.1) |
| **S**ex  Male  Female | 722 (48.2)  776 (51.8) | 67 (50.8)  65 (49.2) |
| **Level of education**  No education  Any education ^a^  School-aged individuals ^b^  Unknown | 767 (51.2)  201 (13.5)  456 (30.4)  74 (4.9) | 35 (26.5)  11 (8.3)  78 (59.1)  8 (6.1) |
| **School-aged individuals’ occupation**  School environment  No work and no school  Work environment | **(N=456)**  413 (90.6)  40 (8.8)  3 (0.6) | **(N=78)**  70 (89.7)  8 (10.3) |
| **Older individuals’ occupation**  Farmers  Other work environment ^c^  No work and no school  School environment  Unknown | **(N=1042)**  487 (46.7)  417 (40.0)  56 (5.4)  8 (0.8)  74 (7.1) | **(N=54)**  22 (40.7)  21 (38.9)  3 (5.6)  0 (0.0)  8 (14.8) |
| **Wealth**  Low  Middle  High | 534 (35.6)  310 (20.7)  654 (43.7) | 46 (34.9)  16 (12.1)  70 (53) |

^a^ Education status includes: unspecified pre-school, kindergarten, primary school, lower and upper secondary school, professional school, college, university and/or above.

^b^ Individuals with school age *i.e.,* aged equal or under-sixteen years old.

^c^ Other work includes: working in a factory, shop, retail industry, hospitality, as laborer, and in an unspecified work environment.

## **Table 4S.** Antibiotic resistance of total cultured *S. pneumoniae* isolates (N=128). MIC breakpoints were defined according to CLSI and EUCAST guidelines.^30,31^ Resistance to 3 or more classes of antibiotics is referred to as multidrug resistance (MDR).^14^

| Antibiotic susceptibility | Total (n, %) |
| --- | --- |
| Susceptible to all antibiotic tested | 25 (19.5) |
| Resistant to at least one drug | 103 (80.5) |
| Non-susceptible to penicillin (PNSP) | 97 (75.8) |
| Resistant to penicillin | 41 (32.0) |
| Resistant to levofloxacin | 0 (0) |
| Resistant to trimethoprim/sulfamethoxazole | 81 (63.3) |
| Resistant to tetracycline | 85 (66.4) |
| Resistant to clindamycin | 99 (77.3) |
| Resistant to erythromycin | 99 (77.3) |
| Multidrug resistance (MDR) | 99 (77.3) |

## **Table 5S**. Univariable logistic analysis of additional risk factors for PNSP carriage in colonized individuals with AST data (N=128). Poultry production was included in the analysis since its derived air pollution has been shown to alter upper respiratory tract microbiota and increase the susceptibility to community-acquired pneumonia.^71^ Ownership of animals, of pigs and of chickens were investigated since *Streptococcus spp.* transmission can occur via contact with nasal or lacrimal secretions of an infected animal and inter- and intra-species horizontal gene transfer (HGT) of mobile genetic elements carrying genes for antibiotic resistance plays an essential role in the evolution of pathogenic pneumococci.^72,73^ HGTs between *S. pneumoniae* and zoonotic pneumococci such as *S. suis* (pigs)^74^ and *S. zooepidemicus* (cattle and chickens)^75^ can lead to the development of antibiotic resistance in susceptible strains. Statistical analyses were performed using sample weights. ABU= antibiotic use.

| **Variable** | **Categories** | **N** | **PNSP (n)** | **PNSP (%)** | **Crude OR [95%CI]** | **p-value** | **Signif.** |
| --- | --- | --- | --- | --- | --- | --- | --- |
| **Sociodemographic** | | | | | | | |
| Sex | Female | 62 | 47 | 75.8 | ref |  |  |
|  | Male | 66 | 50 | 75.8 | 0.93 [0.40-2.17] | 0.863 |  |
| Occupation | Farmers | 23 | 13 | 56.5 | ref |  |  |
|  | Other occupation | 21 | 9 | 42.9 | 0.68 [0.19- 2.36] | 0.541 |  |
|  | School | 66 | 58 | 87.9 | 5.96 [1.85-19.18] | 0.003 | ** |
|  | No job, no school | 11 | 10 | 90.9 | 4.47 [0.47-42.36] | 0.195 |  |
|  | Unknown | 7 | 7 | 100 |  |  |  |
| Adults’ education | Any education | 41 | 18 | 43.9 | ref |  |  |
|  | No education | 5 | 5 | 100 |  |  |  |
|  | Unknown | 1 | 1 | 100 |  |  |  |
|  | School age | 81 | 73 | 90.1 | 11.18 [ 4.09- 30.54] | <0.001 | *** |
| **Host susceptibility to infection** | | | | | | | |
| Seasonality | Spring, summer | 77 | 48 | 62.3 | ref |  |  |
|  | Autumn, winter | 51 | 49 | 96.1 | 11.41 [2.33-55.78] | 0.003 | ** |
| Sampling period | March – April | 64 | 35 | 54.7 | ref |  |  |
|  | Nov. – Dec. | 46 | 44 | 95.6 | 14.38 [2.89-71.55] | 0.001 | ** |
|  | July – Sept. | 18 | 18 | 100 |  |  |  |
| Toilet facility | Septic tank | 122 | 92 | 75.4 | ref |  |  |
|  | Other* | 6 | 5 | 83.3 | 0.98 [0.10-9.21] | 0.986 |  |
| Drinking water  treatment | Only boiled | 109 | 79 | 72.5 | ref |  |  |
|  | Additional treat | 19 | 18 | 94.7 | 4.49 [0.56-35.80] | 0.158 |  |
| Washing hands frequency | Not often | 69 | 45 | 65.2 | 0.22 [0.09- 0.58] | 0.003 | ** |
|  | Often | 59 | 52 | 88.1 | ref |  |  |
| Use of manure | Stool, fertilizer | 19 | 12 | 63.2 | ref |  |  |
|  | Fertilizer only | 27 | 23 | 85.2 | 4.00 [0.94-17.04] | 0.063 |  |
|  | No manure | 82 | 62 | 75.6 | 1.82 [0.60- 5.52] | 0.292 |  |
| Poultry production | 30-80 birds | 33 | 17 | 51.5 | ref |  |  |
|  | <30 birds | 35 | 29 | 82.9 | 4.92 [1.58-15.34] | 0.007 | ** |
|  | No poultry | 60 | 51 | 85 | 3.99 [1.42-11.20] | 0.01 | ** |
| ***S. pneumoniae* development of penicillin resistance/non-susceptibility** | | | | | | | |
| Antibiotic use in animals | No | 54 | 45 | 83.3 | ref |  |  |
|  | Yes | 44 | 31 | 70.5 | 1.22 [0.30-4.95] | 0.781 |  |
|  | Unknown | 30 | 21 | 70 | 1.87 [0.48-7.28] | 0.361 |  |
| Owning animals | Yes | 74 | 52 | 70.3 | ref |  |  |
|  | No | 54 | 45 | 83.3 | 1.53 [0.61-3.82] | 0.365 |  |
| Owning pigs | Yes | 21 | 13 | 61.9 | ref |  |  |
|  | No | 107 | 84 | 78.5 | 2.29 [0.79-6.58] | 0.128 |  |
| Owning chickens | Yes | 67 | 45 | 67.2 | ref |  |  |
|  | No | 61 | 52 | 85.2 | 1.29 [0.55-3.02] | 0.555 |  |
| **Frequency of specific food consumption** | | | | | | | |
| Shrimps | < once per week | 56 | 40 | 71.4 | ref |  |  |
|  | = once per week | 46 | 37 | 80.4 | 1.19 [0.45-3.16] | 0.734 |  |
|  | > once per week | 26 | 20 | 76.9 | 0.99 [0.32-3.06] | 0.988 |  |
| Seafood | < once per week | 56 | 45 | 80.4 | ref |  |  |
|  | = once per week | 37 | 21 | 56.8 | 0.32 [0.12-0.85] | 0.024 | * |
|  | > once per week | 33 | 29 | 87.9 | 1.81 [0.51-6.40] | 0.358 |  |
|  | Unknown | 2 | 2 | 100 |  |  |  |
| Eggs | ≤ once per week | 51 | 37 | 72.5 | ref |  |  |
|  | > once per week | 77 | 60 | 77.9 | 1.20 [0.51-2.83] | 0.674 |  |
| Other meats | < once per week | 89 | 66 | 74.2 | ref |  |  |
|  | = once per week | 20 | 13 | 65 | 0.57 [0.19- 1.67] | 0.307 |  |
|  | > once per week | 16 | 15 | 93.8 | 5.96 [0.73-48.61] | 0.098 |  |
|  | Unknown | 3 | 3 | 100 |  |  |  |

* pit latrine or composting toilet
