# Supplementary material for: Prevalence and determinants of nasal carriage of penicillin non-susceptible Streptococcus pneumoniae: a cross-sectional household survey in northern Vietnam
Source: Lancet Reg Health West Pac. 2025 Jan 8;54:101282. doi: 10.1016/j.lanwpc.2024.101282 (PMC11764309; doi:10.1016/j.lanwpc.2024.101282)
Supplement: Tacoli_PNSP_Abtract_VNese final [file mmc2.docx]

**Cơ sở khoa học**

Beta-lactam vẫn là kháng sinh đầu tay trong điều trị các bệnh nhiễm trùng *Streptococcus pneumoniae* mặc dù tỷ lệ nhiễm các chủng kháng hoặc không còn nhạy cảm với penicillin ngày càng tăng trên toàn cầu. Chúng tôi đã thực hiện một khảo sát cắt ngang các hộ gia đình tại một khu vực nông thôn ở miền Bắc Việt Nam từ 2018 đến 2019 nhằm đưa ra ước tính về tỷ lệ lây truyền *S. pneumoniae* (PNSP) không nhạy cảm với penicillin và để điều tra các yếu tố hành vi và môi trường liên quan đến sự xâm nhập của PNSP. Dữ liệu từ nghiên cứu này sẽ cung cấp thông tin cho việc thiết kế các biện pháp can thiệp vào việc sử dụng kháng sinh không hợp lý tại cộng đồng với quy mô lớn hơn.

**Phương pháp**

Khảo sát được thực hiện từ tháng 7 năm 2018 đến tháng 4 năm 2019, bao gồm 1502 cá nhân từ 324 hộ gia đình. Tổng cộng có 1.498 mẫu từ dịch ngoáy mũi và dịch tỵ hầu được thu thập và được nuôi cấy trên đĩa thạch máu có bổ sung 5 µg gentamicin. Các khuẩn lạc *S. pneumoniae* đã được định danh bằng máy định danh vi sinh vật công nghệ khối phổ (MALDI-TOF). Kiểu hình nhạy cảm với penicilin được thực hiện bằng phương pháp E-test. Các mô hình hồi quy logistic đã được sử dụng để xem xét các yếu tố rủi ro đối với việc mang PNSP so với các chủng nhạy cảm.

**Kết quả**

Chúng tôi đã phân lập được 132 chủng *S. pneumoniae* từ 1.148 mẫu. Kết quả độ nhạy cảm với kháng sinh đạt được là 97% (128/132). Trong số này, 76% (97/128) là PNSP (MIC ≥ 0,12 µg/ml) và 77% (99/128) là không nhạy cảm với 3 loại kháng sinh trở lên. Sau khi điều chỉnh theo độ tuổi và mức sống, việc sử dụng kháng sinh không liên quan đến việc mang PNSP. Trong số những người tham gia khảo sát người trẻ (<20 tuổi) và thường xuyên ăn thịt và các sản phẩm từ sữa có nguy cơ mang PNSP cao hơn , đặc biệt là thịt lợn (OR điều chỉnh 52,30 [KTC 95% 8,72-313,60]) và các chế phẩm từ sữa (OR điều chỉnh 12,48 [4,01-38,82]). Việc tiêu thụ thực phẩm lên men là một yếu tố bảo vệ (OR điều chỉnh 0,02 [<0,01-0,13]).

**Phiên giải**

Tỷ lệ nhiễm PNSP cao nhưng không liên quan đến việc sử dụng kháng sinh của từng cá nhân. Các biện pháp can thiệp ở cộng đồng nhằm giảm tiêu thụ kháng sinh cũng như các nghiên cứu sâu hơn về dư lượng kháng sinh trong các sản phẩm thực phẩm là rất cần thiết để đánh giá vai trò của chúng đối với sự xuất hiện và lan truyền của PNSP.

**Nguồn tài trợ**

Nghiên cứu này được hỗ trợ bởi khoản tài trợ nội bộ của Đơn vị Nghiên cứu Lâm sàng Đại học Oxford từ chương trình Wellcome Trust Châu Phi Châu Á (2015-2022) tại Việt Nam (106680/Z/14/Z).

Disclaimer: This translation in Vietnamese was submitted by the authors and we reproduce it as supplied. It has not been peer reviewed. Our editorial processes have only been applied to the original abstract in English, which should serve as reference for this manuscript.
